# Effects of relaxing breathing paired with cardiac biofeedback on performance and relaxation during critical simulated situations: a prospective randomized controlled trial

**DOI:** 10.1186/s12909-022-03420-9

**Published:** 2022-06-02

**Authors:** Sophie T. Schlatter, Corentin C. Thérond, Aymeric Guillot, Simon P. Louisy, Antoine Duclos, Jean-Jacques Lehot, Thomas Rimmelé, Ursula S. Debarnot, Marc E. Lilot

**Affiliations:** 1 Research on Healthcare Performance (RESHAPE), INSERM U1290, Claude Bernard Lyon 1 University, Lyon, France; 2 Claude Bernard Lyon 1 University, Centre Lyonnais d’Enseignement par Simulation en Santé (CLESS, high fidelity medical simulation center), SAMSEI, Lyon, France; 3 Hospices Civils de Lyon, Departments of Anesthesia and Intensive Care, Lyon, France; 4 University of Lyon, UCBL-Lyon 1, Laboratoire Interuniversitaire de Biologie de la Motricité EA 7424, F-69622 Villeurbanne, France; 5 Hospices Civils de Lyon, Health Data Department, Lyon, France; 6 EA 7426 “Pathophysiology of Injury-Induced Immunosuppression” (Pi3), Université Claude Bernard Lyon, Biomérieux-Hospices Civils de Lyon, Lyon, France; 7 Institut Universitaire de France, Lyon, France

**Keywords:** Biofeedback, Critical situation, Performance, Relaxing breathing, Simulation, Stress

## Abstract

**Background:**

Active participation in high-fidelity simulation remains stressful for residents. Increased stress levels elicited during such simulation impacts performance. We tested whether relaxing breathing, paired or not with cardiac biofeedback, could lead to enhanced performance of residents during simulation.

**Methods:**

This randomized pilot study involved the fifth-year anesthesiology and critical care residents who participated in high-fidelity at Lyon medical simulation center in 2019. Residents were randomized into three parallel interventions: relaxing breathing, relaxing breathing paired with cardiac biofeedback, and control. Each intervention was applied for five minutes immediately after the scenario briefing. The primary endpoint was the overall performance during the simulation rated by two blinded independent investigators. The secondary endpoints included component scores of overall performance and changes in psychological states.

**Results:**

Thirty-four residents were included. Compared to the control group, residents in the relaxing breathing (+ 7%, 98.3% CI: 0.3 to 13.7, *P* = 0.013) and relaxing breathing paired with cardiac biofeedback (+ 8%, 98.3% CI: 0.82 to 14.81, *P* = 0.009) groups had a higher overall performance score. Following the interventions, compared to the control group, stress level was lower when participants had performed relaxing breathing alone (*P* = 0.029) or paired with biofeedback (*P* = 0.035). The internal relaxation level was higher in both the relaxing breathing alone (*P* = 0.016) and paired with biofeedback groups (*P* = 0.035).

**Conclusions:**

Performing five minutes of relaxing breathing before the scenario resulted in better overall simulation performance. These preliminary findings suggest that short breathing interventions are effective in improving performance during simulation.

**Trial registration:**

The study protocol was retrospectively registered on clinicaltrials.gov (NCT04141124, 28/10/2019).

**Supplementary Information:**

The online version contains supplementary material available at 10.1186/s12909-022-03420-9.

## Introduction

Healthcare professionals dealing with emergencies and critical care regularly experience sudden feelings of acute stress which can be associated with performance deterioration in both technical and non-technical skills [1–4]. Stress management techniques (SMT) have been reported as effective approaches to reduce the intensity of the stress reaction. Regular training in SMT was further reported to reduce sleep disorders, burnout and depression, all long-term stress-related effects [5, 6]. Spurred by these benefits, routine use of SMT was proposed to help healthcare practitioners dealing with stressful critical events. However, the impact of acute interventions remains less explored [6–8]. In many clinical situations, stress can be predicted before the occurrence of the actual stressor (e.g., expecting a new patient in a context of bed saturation, anticipated arrival of an emergency). The early identification of the subsequent stressor leads to a period of anticipatory stress. During this anticipatory period a preventive coping method might be applied in order to decrease the subsequent stress response  [9, 10], with the intention of improving performance during the following critical events.

Breathing exercises are a core element of relaxation techniques that could be used as SMT. Breathing can be slowed down to a constant regular rate leading to a respiratory sinus arrhythmia that contributes to a shift towards the parasympathetic central nervous system, increasing relaxation, and reducing stress levels [11]. Providing information on physiological responses is a promising alternative to reduce physiological and psychological stress indicators [9, 10, 12, 13]. In healthy participants, cardiac biofeedback training leads to a reduction in self-reported stress and anxiety [14]. Additionally, recent studies reported that acute use of such techniques might help to maintain optimal cognitive abilities [10, 15]. However, the acute effects of these techniques on performance, in a clinical professional stress context, remain unexplored. Simulation offers an interesting immersive and realistic context, for studying the effects of a SMT before its implementation in clinical practice, without exposing patients to risk.

High-fidelity simulation has been formally implemented in the curriculum of residents to improve their performance, notably in critical clinical situations, through the development of technical and non-technical skills [16–18]. However, simulation can also induce high stress levels, impacting these skills during the scenario [2, 3, 19, 20]. The benefit of a short period of relaxing breathing paired with biofeedback on the overall performance and stress level experienced during high-fidelity simulation remains unexplored. The main hypothesis was that five minutes of proactive relaxing breathing, with or without concurrent biofeedback, performed prior to the scenario would improve performance during simulated critical care situations. We also hypothesized that these short interventions may reduce the anticipatory stress induced before the simulation. The cardiac biofeedback was expected to potentiate the effect on relaxation.

## Material and Methods

### Ethics approval / license / Registration

The study protocol was approved by the Institutional Review Board of Claude Bernard University Lyon 1, Lyon, France (n°IRB 2019_07_09_03, July 2019) and informed consent was obtained from all subjects before participating in the trial. The research was performed in accordance with the Declaration of Helsinki. The trial was retrospectively registered at clinicaltrials.gov (Marc Lilot, NCT04141124, Date of registration: 28/10/2019). The results were reported using the Consolidated Standards of Reporting Trials guidelines [21].

### Population and setting

This study involved all fifth-year anaesthesiology and critical care residents from Lyon University who participated in high-fidelity simulation at the Lyon medical simulation centre in 2019. No exclusion criteria were applied. Simulations were part of the resident’s educational program and each session lasted four to five hours. Each session was composed of the same four different scenarios. Each resident participated individually in one scenario. Simulations were always structured as follows: briefing (one to five min), scenario (10 to 20 min), and debriefing (30 to 45 min) [22–24]. The scenarios dealt with crisis situations in the intensive care unit, operating room, and delivery room (Tamponade, Neonatal, Amniotic Fluid, Pacemaker), no residents performed these scenarios beforehand (App. A). For each scenario, the instructor playing the embedded nurse acted as neutrally as possible. SimMan Essential® and SimNewB® manikins (Laerdal Medical AS, Stavanger, Norway) were used.

### Design

**Fig. 1:**
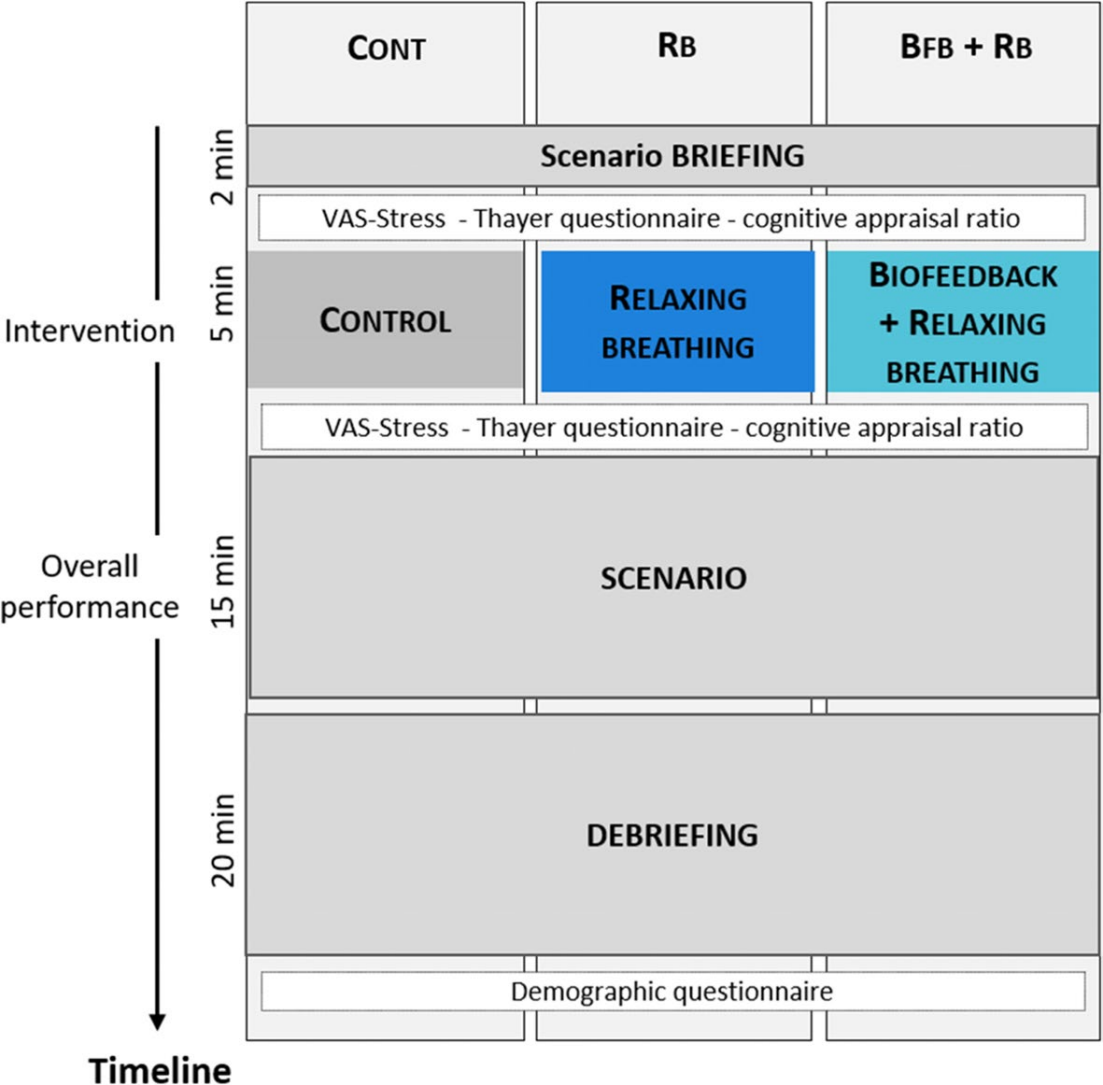
Timeline of the experimental design. VAS: Visual Analogue Scale. The resident received the briefing of the scenario first, followed by the intervention, the scenario, and the debriefing. The breathing intervention consisted of a relaxing breathing exercise (iterative sequence of 4 s of inspiration and 6 s of expiration). The biofeedback + relaxing breathing intervention corresponded to the relaxing breathing exercise paired with the viewing of real-time cardiac parameters. Overall performance corresponded to both technical (clinical specific evaluation grid) and non-technical skills (Ottawa scale) performance

This prospective randomized controlled study involved three parallel arms and a hypothesis of superiority (1:1:1 allocation). Two sessions of simulation were performed daily (one in the morning and one in the afternoon). A blinded investigator assigned each session of simulation to one intervention (Fig. 1) (Rb, Bfb + Rb, Control).

Then, following simple computerized randomization procedures, residents were randomly allocated to a session of simulation (M.L.). Upon arrival, residents were informed about the study and told that the research was interested in well-being during simulation (M.L./S.S.). They were briefly informed that they will perform a breathing exercise at various times during the session of simulation (before the scenario or after the debriefing). Participants were blinded to working hypotheses and randomization procedures. Each intervention was performed by the main investigator (S.S) in an isolated room immediately after the briefing, so that all instructors leading the simulation were blinded to the group allocation. The active participant sat on a chair, while the investigator stayed in the room and verified that residents performed their interventions.

The three interventions were:

A) Rb: Residents in the relaxing breathing (Rb) group received standardized relaxing breathing. The resident was asked to follow, for five minutes, a standardized rhythm of breathing, with an inspiration for four seconds and expiration of six seconds. The standardized relaxing breathing was guided by looking at a moving breathing cursor on a computer. No visual cardiac biofeedback was provided.
B) Bfb + Rb: The residents in the heart rate variability biofeedback paired with relaxing breathing group (Bfb + Rb), were asked to follow the same standardized breathing as in the Rb group. The standardized relaxing breathing was guided by looking at a smartphone screen (Iphone 5S ™, Apple, cupertino, CA, USA) and a heart rate variability-biofeedback was provided on the same screen through a connection to a cardio frequency meter placed on the resident’s ear lobe (Stress control™, My Mercurochrome®, Paris, France). Through the help of the interface, residents were asked to try to increase their heart rate variability.
C) Control: Residents from the control group reviewed normal printed laboratory test results. They were informed that those tests were unrelated to the scenario. The resident was asked to read the results for five minutes. This control has been used in previous studies, and might be seen as a normal standardized clinical practice [25–27].

Each intervention was conducted in a standardized manner for 5 min. Afterwards, the participant went directly to the simulation room and the scenario started. All had received a formal training in Tactics to Optimize the Potential in 2017 [25]. These tactics combined specific tools of mental preparation such as mental imagery and projection of success with cognitive toolboxes.

### Performance evaluation

Two assessors (M.L./C.T.) blinded to group allocation evaluated performance independently using video recordings. For each scenario, a checklist was established beforehand to assess specific aspects of clinical performance. Each item on the checklist was associated with a number of points so that the total reached 100. These checklists have been extensively described previously [26, 27]. For each scenario, the Ottawa Crisis resource management Global Rating Scale (Ottawa GRS) was used to assess non-technical skills. Ratings for the six criteria were summed, scores ranging from 6 to 42 points [28]. Then, for each resident, the average of the two assessors’ scores for each performance (clinical specific or non-technical skills) was computed.

### Questionnaires

Before and after the intervention, each resident completed a 10 cm Visual Analogue Scale (VAS) of stress (VAS-Stress), participants also answered the Thayer questionnaire (Activation-Deactivation Adjective Check List) [29]. The Thayer questionnaire is a multidimensional questionnaire of transitory arousal states [5 to 20 points], including energetic (activation, deactivation) and tense (tension, relaxation) arousals. All residents filled out a questionnaire on demographic data and the BIG-5 personality inventory assessing 5 dimensions of personality (Openness, Consciousness, Extraversion, Agreeableness, Neuroticism) [30]. Neuroticism is known to confer a particular vulnerability to stress [31]. At the end of the experiment, all residents judged if their intervention could be used in their professional or personal practice (VAS use 10 cm, Supplemental Fig. 1).

### Endpoints

The primary endpoint was the mean overall performance during simulation. This endpoint was calculated as the sum of the clinical performance score out of 100 points plus the Ottawa GRS score (6 to 42) adjusted to a scale of 100 points (score /42*100). The sum was then divided by two to obtain an overall score between zero and 100 points. Secondary endpoints were clinical performance, Ottawa GRS scores, VAS-Stress, Thayer questionnaire scores (relaxation, tension, activation, deactivation).

### Statistical analyses

**Fig. 2:**
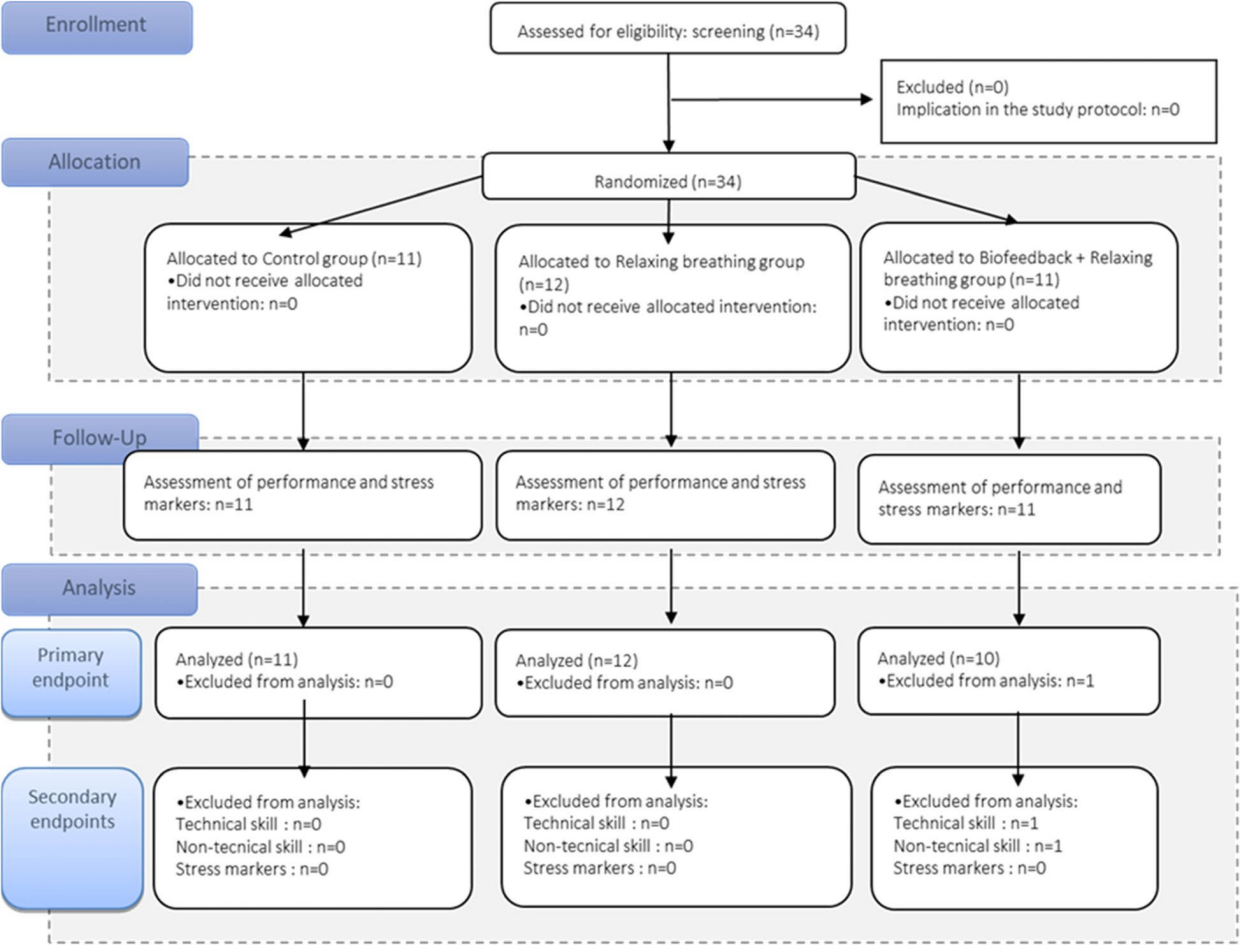
The study flow chart described this prospective randomized controlled study involved three parallel arms and a hypothesis of superiority (1:1:1 allocation). The figure follows the guidelines of Moher et al. 2010 (Moher et al., CONSORT explanation and elaboration: updated guidelines for reporting parallel group randomised trials. *BMJ,* 2010)

The Consolidated Standards of Reporting Trial is presented in Fig. 2. Statistical analyses were performed with R studio version 1.2.1335 (R Foundation, Vienna, Austria). All tests were two-tailed. Visual assessments were used to confirm normality of data distribution with histograms and quantile-quantile plots. For all performance measures, inter-rater reliability of investigators was assessed by calculating absolute interclass correlation coefficients and 95% CI for individual measures (package DescTools, ICC function); agreement was interpreted according to Cichetti [32]. We assessed the treatment effect on performance (overall, technical skills and non-technical skills) using an analysis of variance including the main effects of group (Rb, Bfb + Rb, Control) and scenario, and assessing the group-by-scenario interaction. Results are presented as differences in mean between groups with 98.3% confidence interval (CI) and P < 0.017 was the significance criterion when the 3 groups were compared. An outlier detection test was first performed on all variables of performance (± 1.5 inter-quartile range rejection threshold). Effect sizes are reported using eta squared (η^2^). The effect size can be classified as small (0.01), medium (0.06) or large (> 0.14). We assessed the effects of the group (Rb, Bfb + Rb, Control) on psychological variables (VAS-Stress, Thayer scores) using linear regression controlled for the scenario, the basal level, the level of neuroticism. Normality of residuals of the models were checked. Results are presented as estimate (standard error). For the multiple regression, the adjusted R^2^ was provided. No data was available to calculate a priori the sample size requested. Therefore, this pilot study included all the fifth-year anaesthesiology and critical care residents who participated in high-fidelity simulation at the Lyon medical simulation centre in May 2019 in order to assess the interest for a further study allowing a deeper exploration of the psychophysiological effects of these stress management techniques.

## Results

A total of 34 residents was included in this analysis (requitement and follow up during May 2019) (Supplemental Table 1). No resident declined to participate. Characteristics did not differ between groups (Table 1). Outlier detection led to the exclusion of one participant for the overall performance and the Ottawa GRS performance (Supplemental Fig. 2). For each performance score, means ± SD are presented (Table 2).

**Table 1:** Characteristics data of residents receiving Control, Relaxing Breathing (Rb), or Relaxing Breathing with Biofeedback (Bfb + Rb) intervention. Values expressed as n or mean ± SD

	Control *n*=11	Rb *n*=12	Bfb + Rb *n*=11
Characteristic data			
Female, n	5	6	3
Age, years	29 ± 1	29 ± 1	29 ± 1
Previous simulation, n	7 ± 2	6 ± 2	7 ± 2

**Table 2:** Performance scores of residents receiving Control, Relaxing Breathing (Rb), or Relaxing Breathing with Biofeedback (Bfb + Rb) intervention. Values expressed as mean ± SD

	Control	Rb	Bfb + Rb
Overall performance /100	64 ± 7	71 ± 10	72 ± 6
Technical skills (Clinical specific performance) /100	48 ± 11	58 ± 13	55 ± 19
Non-technical skills (Ottawa scale performance) /42	34 ± 3	36 ± 2	35 ± 4

### Primary endpoint

**Fig. 3:**
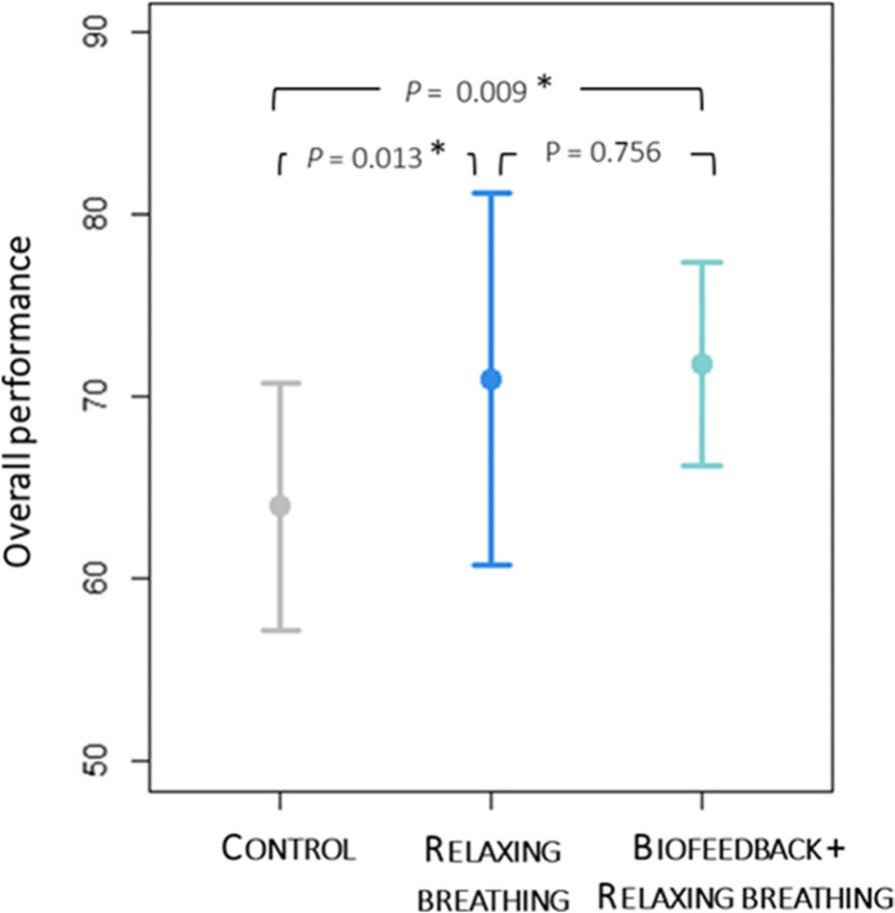
Overall performance scores during scenarios of high-fidelity simulation. Points and arrows represent means and standards deviations. We assessed the treatment effect on the primary endpoint (overall performance) using linear regression model including main effects of group (Rb, Bfb + Rb, Control) and scenario, and assessing the group-by-scenario interaction. *P* < 0.017 was the significance criterion when there were 3 groups being compared

Overall performance was higher in the Rb compared with the Control group (Fig. 3, difference: 6.98 (98.3% CI [0.30 to 13.67], *P* = 0.013). There was also a higher performance in the Bfb + Rb group compared with the Control group (7.82 [0.82 to 14.81], *P* = 0.009). No difference was observed between  Bfb + Rb and Rb groups (0.83 [-6.02 to 0.756], *P* = 0.756). A main effect of scenario (*P* = 0.016, η^2^ = 0.21) and a scenario × group interaction (*P* = 0.045, η^2^ = 0.26) were identified. However, no significant specific interactions remained after correcting for multi-testing.

### Secondary endpoints

#### Performances

##### Technical skills: Clinical specific performance

The absolute interclass correlation coefficient for assessment of clinical specific performance was excellent (0.98, 95% CI: 0.96 to 0.99). Rb had higher clinical specific performance than in the Control group (10 [1.00 to 19.91], *P* = 0.009). Bfb + Rb had no statistical score difference with the Control group (7 [-2.61 to 16.70], *P* = 0.073), however a trend (*P* < 0.10) for higher score in the Bfb + Rb was detected. No difference between Bfb + Rb and Rb groups’ scores was found (-3.41 [-12.87 to 6.04], *P* = 0.361). A significant effect of scenario (*P* < 0.001, η^2^ = 0.45) was found.

##### Non-technical skills: Ottawa scale performance

The absolute interclass correlation coefficient for the assessments of Ottawa scale performance was good (0.67, 95% CI: 0.48 to 0.80). There was no group difference (*P* = 0.285), no significant effect of scenario (*P* = 0.942), and no scenario × group interaction (*P* = 0.846).

### Stress level

**Fig. 4:**
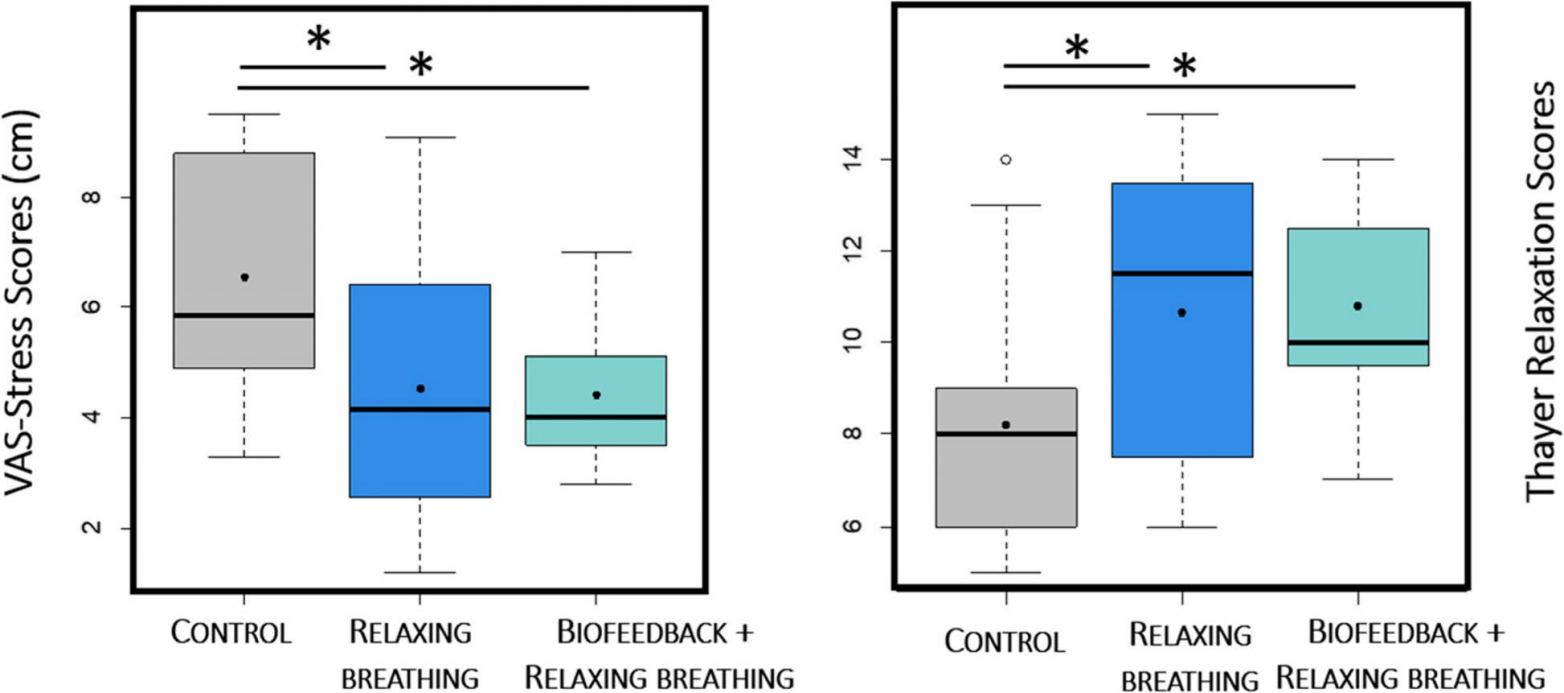
Psychological stress after the intervention. VAS-Stress: Visual analogous scales for stress. The points indicate the means. The grey boxplots indicate the control group, the dark blue indicate the relaxing breathing group and the turquoise ones indicate the biofeedback + the relaxing breathing group

The linear model of stress (adjusted R^2^ = 0.22) revealed that VAS-Stress scores in both interventional groups were lower than in the control group (Fig. 4, Control vs Rb, -2.02 ± 0.89, *P* = 0.029) (Control vs Bfb + Rb, -2.02 ± 0.86, *P* = 0.035). The score did not differ when comparing both interventional groups (Rb vs Bfb + Rb, *P* = 0.897).

The linear model of relaxation (adjusted R^2^ = 0.41) showed that both interventional groups reported higher scores than the control group (Fig. 4, Control vs Rb, 2.80 ± 1.09, *P* = 0.017; Control vs Bfb + Rb, 2.41 ± 1.08, *P* = 0.035). The relaxation did not differ between interventional groups (*P* = 0.709). The linear model of tension (*P* = 0.025, R^2^ = 0.2751) showed that values reported in the interventional groups did not differ from those reported in the control group (Control vs Rb, *P* = 0.275 and Control vs Bfb + Rb, *P* = 0.213). The tension did not differ between interventional groups (*P* = 0.875).

The linear model of activation (adjusted R^2^ = -0.03) revealed that interventional groups did not differ from the control group (Control vs Rb, *P* = 0.427 and Control vs Bfb + Rb, *P* = 0.632). The activation did not differ between the two intervention groups (*P* = 0.183). The linear model of deactivation (adjusted R^2^ = 0.25) showed no difference between groups (Control vs Rb, *P* = 0.521) (Control vs Bfb + Rb, *P* = 0.341) (Rb vs Bfb + Rb, *P* = 0.099).

## Discussion

This pilot study firstly showed that an acute short session of relaxing breathing, alone or paired with cardiac biofeedback, used as a stress management intervention improved the overall performance of residents during simulation. Secondly, the results showed greater decrease of stress and increase in relaxation of participants in the two stress management intervention groups compared with the control group. Taken together, these results indicate that relaxing breathing, paired or not with biofeedback, might contribute to help residents to cope with the anticipation stress of simulated critical situations. Therefore, subsequent simulation performance might benefit from the implementation of short proactive coping methods through mastery of physiological regulation processes based on standardized breathing. Further studies need to confirm the results observed here and to explore whether there is any improvement in real subsequent performance after application of stress management training.

The improvement of overall performance seems supported by the enhancement of technical skills, notably following relaxing breathing. This improvement might have a positive impact on patient outcomes which is the ultimate objective in simulation. Medical technical skills are supported by memorized explicit knowledge, flexibility, and inhibitory control. As previous studies reported that these cognitive abilities are especially affected by higher stress levels, the decrease in stress induced by the SMT may have prevented the deleterious effects on cognitive performance [4]. Previous findings in a laboratory acute stress context showed that relaxing breathing paired with biofeedback contributed to improved reaction time and/or the accuracy of a cognitive task which relied on updating working memory, mental set shifting, and inhibition abilities [10, 15]. The interventions could have helped with the preservation of these abilities resulting in higher technical performance.

As opposed to technical performance, present results suggest that non-technical performance was not influenced by breathing interventions. Non-technical skills refer to resources that are now increasingly considered to complement technical skills, contribute to relevant and efficient task performance and to minimize adverse events during patient care [33, 34]. Previous SMT studies, such as Tactics to Optimize Potential, reported positive impacts on non-technical skills performance [26, 27]. The tactics were acquired over five weeks and were specifically reactivated just before the scenario (e.g., mental dynamization followed by one minute of revitalizing breathing) [26]. In contrast, the breathing exercise performed in the present study lasted 5 minutes and was relaxing. Therefore, the type of breathing should be further explored for its potential to produce different effects on both technical and non-technical skill performance during critical care simulations.

Previous studies reporting positive effects of stress management techniques on performance during simulation were based on protocols including frequent practice (from three days to five weeks), with an additional reactivation phase [26, 35]. Long training including regular exercises presents important limits, as adherence and frequency of practice might be highly heterogeneous between individuals, and learning remains resource-intensive and time-consuming. Recent findings in a laboratory acute stress context showed that 15 minutes of relaxing breathing paired with biofeedback contributed to decreased psychophysiological anticipatory stress and improved perfromance [9, 10]. Here, a very short session (five-min) of relaxing breathing interventions demonstrated favourable effects on performance and relaxation. The enhancement of performance might result from improvement in emotional regulation before and during the scenario [20]. The present results support the hypothesis that really short interventions might contribute to help residents to cope with the anticipated stress of critical situations.

The 5-min relaxing breathing exercise, paired or not with biofeedback, is quick, easy to use and applicable to many standard clinical situations without delaying care. After a prior short and standardized training, relaxing breathing can be performed before any expected critical situation resulting in anticipatory stress such as waiting for a polytraumatized patient, facing a difficult situation in obstetrics or before dealing with an expected difficult airway management. In fact, one can imagine the applicability of this SMT alone or collectively, when five minutes are available before delivering appropriate care. Furthermore, the implementation of SMT into the curriculum of residents should increase its subsequent use in clinical practice. Still, it should be noted that some of the scenarios explored here involved crisis resource management for which in real life there is no time available to anticipate and carry out a relaxation exercise. Further studies should explore these interventions during a period of clinical anticipatory stress and determine the influence or shorter exercises. The effect and applicability of relaxing breathing to real clinical situations should be further explored.

The present study did not show any further improvement with the additional cardiac biofeedback. Biofeedback was expected to improve adherence of the practitioner through awareness of the physiological effects [12–14]. It is possible that the biofeedback potentiation of relaxing breathing might be restricted to physiological components of stress. Additional studies assessing both the physiological parameters and the long-term biofeedback practice, should help to clarify the putative additional effect of biofeedback.

This pilot study has some limitations. While reading biological test results has been extensively used as a control in previous studies, it might also generate extra cognitive load and/or an attention bias [25–27]. A larger sample size might have allowed exploration of psychometric or experiential factors associated with effects of relaxing breathing and biofeedback. As the simulation sessions were long and the order of scenarios was not randomized, fatigue could be a potential cause of the scenario-intervention interaction identified. Therefore, the influence of fatigue on effectiveness of SMT should be further explored. Second, no assessment of baseline clinical performance was performed before simulation. However, the homogeneity of training undertaken by the end of a residency at the same university hospital, associated with randomization should have reduced this potential bias. Third, the effect observed in subjective psychological data remains small. Stress level could have been measured with multisource analyses, including physiological samples, to extensively explore the impact of interventions on stress levels. Finally, no assessment of training in daily stress coping practices was collected. Further studies should also consider individual knowledge, regular practice of stress coping techniques (e.g., yoga, meditation) and assess whether residents used additional techniques during the simulation. One might suspect that experimentation of stress management techniques during the curriculum of residents should initiate their subsequent use in clinical practice. Assessing the long-term effects of these short breathing interventions on both the future spontaneous use of SMT and long-term memory of educational messages should be further explored as the principal objective of a study.

## Conclusion

Performing a proactive 5-min period of stress management with relaxing breathing alone (+7%) or paired with biofeedback (+8%) leads to an increase in overall performance during high-fidelity simulation. Deeper analysis on the two components of performance score revealed that the overall improvement results mostly from an increase in technical skills. This pilot study provides evidence for the benefit of an acute short intervention of breathing, paired or not with cardiac biofeedback, on stress reduction and relaxation improvement prior to simulation of critical situations. We suggest that this anxiety reduction may have protected the performance of residents from being negatively impacted by their stress level. Visualizing cardiac biofeedback did not seem to add an additionnal benefit to the relaxing breathing in this setting.

## Supplementary Information

**Additional file 1.**

**Additional file 2.**

**Additional file 3.**

## Data Availability

The datasets used and/or analysed during the current study are available from the corresponding author on reasonable request.

## Abbreviations

SMT: Stress management technique
Rb: Relaxing breathing
Bfb + Rb: Heart rate variability biofeedback paired with relaxing breathing group
Ottawa GRS: Ottawa crisis resource management global rating scale
VAS: Visual Analogue Scale
CI: Confidence interval
SD: Standard deviation
