# Supplementary material for: Effects of relaxing breathing paired with cardiac biofeedback on performance and relaxation during critical simulated situations: a prospective randomized controlled trial
Source: BMC Med Educ. 2022 Jun 2;22:422. doi: 10.1186/s12909-022-03420-9 (PMC9164463; doi:10.1186/s12909-022-03420-9)
Supplement: Supplementary file 1 — Additional file 1. [file 12909_2022_3420_MOESM1_ESM.docx]

**Appendices**

**Appendix A: Simulated scenarios and related briefings**

| **Scenario** | **Topic** | **Summary of the briefing** |
| --- | --- | --- |
| **Tamponade** | Intra hospital cardiac arrest caused by hemopericardium leading to tamponade and sudden cardiac arrest | You are called to see a man with acute dyspnea and hypotension occurring few days after cardiac surgery |
| **Neonatal cardiac arrest** | Desaturation and neonatal severe bradycardia immediately after childbirth | You are called for neonatal desaturation in the delivery room |
| **Amniotic fluid embolism** | Amniotic fluid embolism leading to cardiac arrest of a pregnant woman immediately before non-urgent caesarean section | You are going to care for a pregnant woman for a non-urgent caesarean section |
| **Pacemaker dysfunction** | Pacemaker and implantable cardioverter defibrillator dysfunction during emergency surgery using electrocautery | You are supervising emergency general anaesthesia in a patient with pacemaker and implantable cardioverter defibrillator |
