# Supplementary material for: Effects of relaxing breathing paired with cardiac biofeedback on performance and relaxation during critical simulated situations: a prospective randomized controlled trial
Source: BMC Med Educ. 2022 Jun 2;22:422. doi: 10.1186/s12909-022-03420-9 (PMC9164463; doi:10.1186/s12909-022-03420-9)
Supplement: Supplementary file 2 — Additional file 2. [file 12909_2022_3420_MOESM2_ESM.docx]

**Highlights:**

- High-fidelity simulation of critical care generates a high level of stress
- Stress level of participants might affect their performance during simulation
- Relaxing breathing and biofeedback are efficient stress management techniques
- 5-min of relaxing breathing and biofeedback resulted in better overall performance
- Relaxing breathing should be tested during anticipated clinical emergencies
