# Supplementary material for: Effects of relaxing breathing paired with cardiac biofeedback on performance and relaxation during critical simulated situations: a prospective randomized controlled trial
Source: BMC Med Educ. 2022 Jun 2;22:422. doi: 10.1186/s12909-022-03420-9 (PMC9164463; doi:10.1186/s12909-022-03420-9)
Supplement: Supplementary file 3 — Additional file 3. [file 12909_2022_3420_MOESM3_ESM.docx]

**Supplemental Material**

**Supplemental Figure 1: VAS use.**

All residents at the end of the experiment judge if their intervention method could be used in their professional or personal practice. They had to answer this question on a 10 cm VAS, ranging from absolutely not to completely. The points indicate the means. The grey boxplots indicate the control group, the dark blue indicate the relaxing breathing group and the turquoise ones indicate the biofeedback + the relaxing breathing group. No difference between groups was detected (χ2 (2) =3.16, P = 0.324).

**Supplemental Table 1: Simulated scenarios and group repartition**

Intervention group: Control, relaxing breathing (Rb), or relaxing breathing with biofeedback (Bfb + Rb).

**Supplemental Figure 3: Outliers detection for the overall performance (± 1.5 Inter Quartile Range (IQR) method).**

**Note.** The exclusion of outliers is conventional in studies interesting the impact of stress/anxiety and stress management intervention on performance (e.g. Pulopulos et al. 2020, Telzer et al. 2008, Ioannou et al. 2016).

**Supplemental Table 2: Performance scores (points, mean ± SD).**

Intervention group: Control, relaxing breathing (Rb), or relaxing breathing with biofeedback (Bfb + Rb).


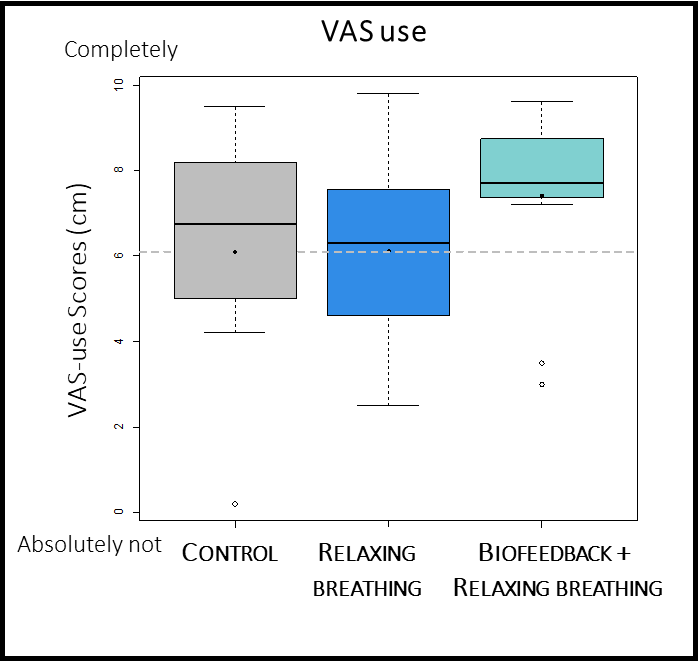
**Supplemental Figure 1: VAS use.**

**Supplemental Table 1: Simulated scenarios and group repartition**

|  | **Control** | **Rb** | **Bfb + Rb** |
| --- | --- | --- | --- |
| **Tamponade** | 2 | 3 | 3 |
| **Neonatal cardiac arrest** | 3 | 4 | 3 |
| **Amniotic fluid embolism** | 2 | 4 | 2 |
| **Pacemaker dysfunction** | 4 | 1 | 3 |

**Supplemental Figure 2: Outliers detection for the overall performance (± 1.5 Inter Quartile Range (IQR) method).**

**
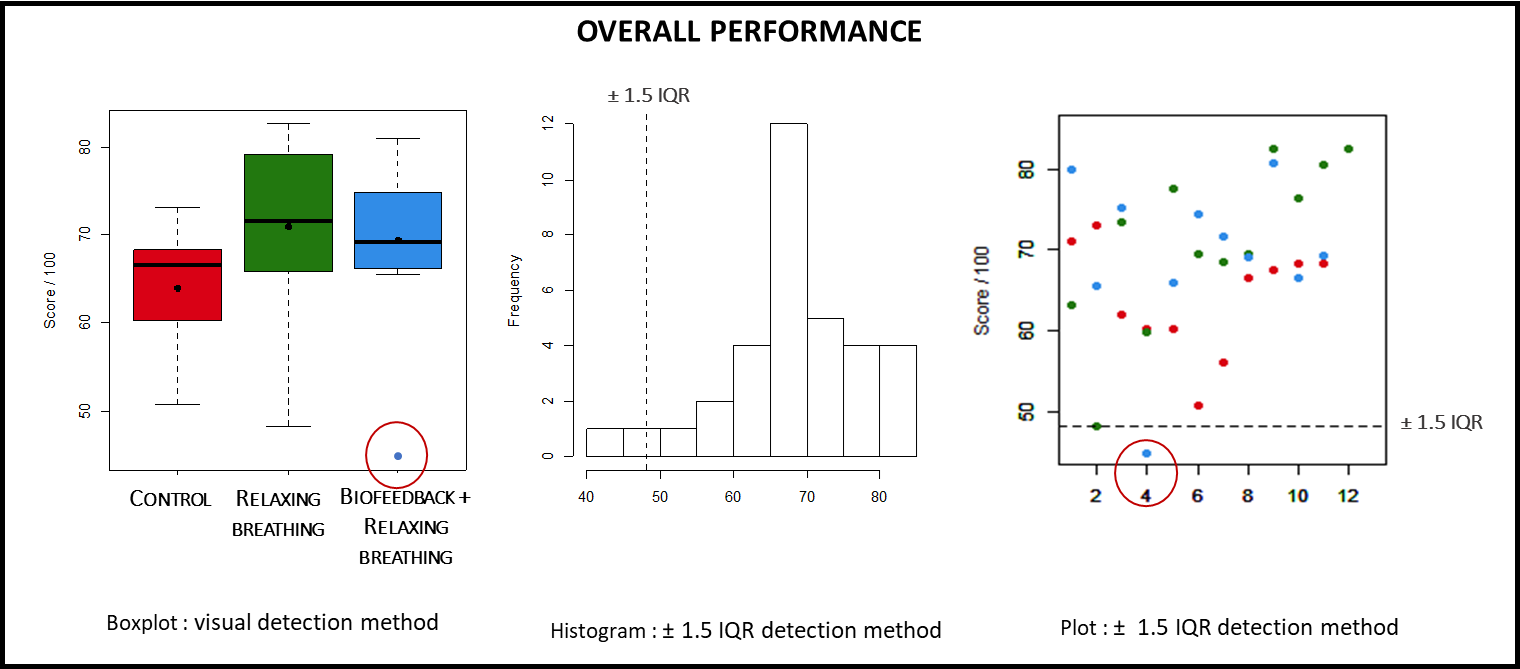
**
